# Antibacterial Activity and Mechanism of Self-Assembly Spermidine-Capped Carbon Dots against *Staphylococcus aureus*

**DOI:** 10.3390/foods13010067

**Published:** 2023-12-23

**Authors:** Tianqi Cui, Ya Fan, Yaping Liu, Xuejing Fan, Yuxue Sun, Guiguang Cheng, Jianjun Cheng

**Affiliations:** 1 Faculty of Food Science and Engineering, Kunming University of Science and Technology, Kunming 650550, China; 2 College of Food Science, Northeast Agricultural University, Harbin 150030, China

**Keywords:** carbon dots, antibacterial mechanism, *S. aureus*, membrane damage, ROS generation

## Abstract

This paper investigated the antibacterial mechanism of spermidine-capped carbon dots (S-PCDs) against *Staphylococcus aureus*. The results showed that there were a large number of amino groups on the surface of S-PCDs and they had a high positive charge (+47.06 mV), which could be adsorbed on the negatively charged bacterial surface through electrostatic interaction and changed the permeability of the bacterial cell membrane. The extracellular protein and nucleic acid contents of *S. aureus* treated with S-PCDs were 5.4 and 1.2 times higher than those of the control group, respectively. The surface folds and defects of the bacterial cell membrane, and the leakage of cell contents were observed using SEM and TEM. The expression of metabolic oxidation regulatory genes *dmpI*, *narJ* and *narK* was upregulated and the intracellular ROS generation was induced, causing bacterial oxidative stress and eventually bacterial death. S-PCDs can effectively inhibit biofilm formation and had low cytotoxicity. The S-PCD treatment successfully inhibited microbial reproduction when pasteurized milk was stored at 25 °C and 4 °C. These results provide important insights into the antimicrobial mechanism of S-PCDs and lay the foundation for their application in the food field as a potentially novel bacteriostatic nanomaterial.

## 1. Introduction

Food safety is one of the main concerns of the global health and food industry [1]. Foodborne diseases caused by foodborne pathogens account for the highest proportion of food safety incidents [2]. *Staphylococcus aureus* is a classic gram-positive pathogen that produces seven different toxins that are common causes of foodborne illness [3]. *S. aureus* can contaminate milk and its dairy products at all stages of production, processing and storage, leading to foodborne illness and major safety concerns [4]. At present, nanoparticles (NPs) have been widely used as antimicrobial agents in the food field due to their ability to effectively inhibit pathogenic microorganisms’ growth [5,6]. However, most NPs are potentially toxic [7]; therefore, the development of safe NPs with high antibacterial activity to inhibit *S. aureus* in food is essential.

Carbon dots (CDs) are a novel carbon nanomaterial with a size below 10 nm, which have been widely used in many fields due to their potential for a wide range of technological applications [8]. The size, surface charge and surface functional groups of CDs affect their antibacterial properties. The surface chemical modification of CDs by specific groups can cause strong interaction between CDs and bacterial cells, thus seriously damaging bacterial cell membranes [9]. In addition, CDs can induce elevated ROS levels which can disrupt normal bacterial metabolism by damaging DNA, proteins and enzymes in cells [10]. Wu et al. (2022) synthesized levofloxacin-based carbon spots (LCDs) using the one-pan hydrothermal method. LCDs have an effective antimicrobial property and destroy bacteria based on the dual antibacterial mode [11]. Different raw materials have great influence on the antibacterial properties of CDs. Polyethyleneimine (PEI) is a kind of compound with antibacterial activity. In addition, the characteristics of polycation, high charge and biocompatibility make spermidine suitable for surface modification of NPs [12]. Azevedo et al. (2014) used PEI to synthesize CDs with anti-biofilm and antibacterial activity against *S. aureus* and other bacteria [13]. Li et al. (2016) adopted a two-step method to synthesize spermidine-based fluorescent carbon quantum dots (SPD-CQDs), which showed strong antibacterial activity against *S. aureus* multidrug-resistant *E. coli* and methicillin-resistant *S. aureus* [14]. In addition to the advantages of simple synthesis method, low cost, good dispersion, controllable surface chemical structure, etc., CDs also have low toxicity and high biocompatibility, which makes CDs an antibacterial material widely used in the field of smart food packaging.

In this study, spermidine-capped carbon dots (S-PCDs) were synthesized using a hydrothermal reaction, and their morphologies and chemical structures were characterized by TEM, FT-IR, XPS and other technologies. The aim was to assess the antibacterial activity, antibacterial mechanism and antibiofilm formation activities of S-PCDs against *S. aureus*. The cytotoxicity of S-PCDs was also examined. Furthermore, the S-PCDs were applied to the storage of pasteurized milk at 25 °C and 4 °C, respectively, to explore their feasibility as additives in food production. The aim of this work is to provide a new effective method for the control of *S. aureus* in the food field.

## 2. Materials

Spermidine (99%) and kanamycin were purchased from Yuanye Biotechnology Co., Ltd. (Shanghai, China). Polyethyleneimine (PEI_10000_) was purchased from Macrlin Biochemical Technology Co., Ltd. (Shanghai, China). NCTC L929 fibroblast cells and L929 medium, Calcein/PI Cell Viability/Cytotoxicity Assay Kit and Cell Counting Kit-8 (CCK-8) were purchased from Procell Life Science & Technology Co., Ltd. (Wuhan, China), Beyotime Biotechnology (Shanghai, China) and Labgic Technology Co., Ltd. (Beijing, China). Live/Dead BacLight Bacterial Viability Kit (L7012) was purchased from Thermo Fisher Scientific (Waltham, MA, USA).

### 2.1. Staphylococcus aureus Culture

*S. aureus* is a strain isolated and purified from rotten turbot in our laboratory. The *S. aureus* was cultured in LB medium using the method of Liu et al. (2022) [15]. Approximately 1 × 10^8^ CFU/mL of bacterial suspension was obtained after 24 h culture at 37 °C for further experiments.

### 2.2. Synthesis of S-PCDs

**Figure 1:**
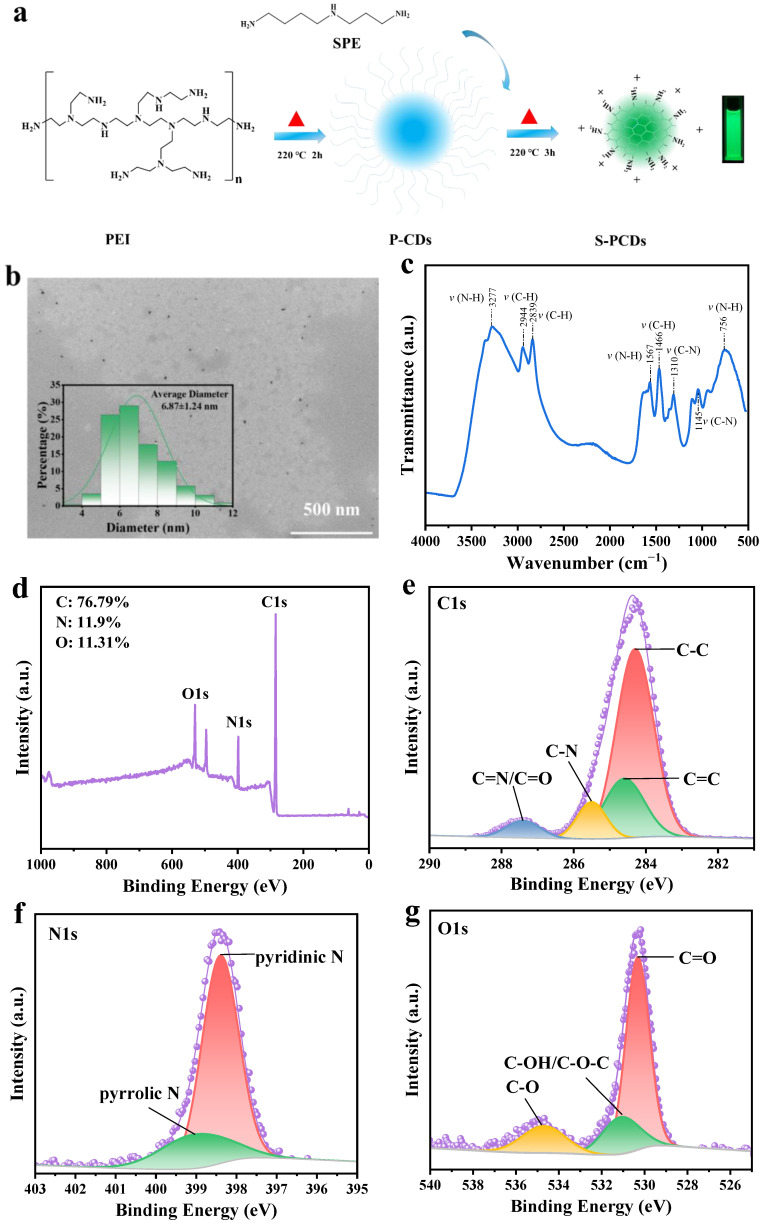
Structure characterization of S-PCDs. (**a**) Hydrothermal reaction synthesized spermidine-capped carbon dots (S-PCDs). (**b**) TEM images and particle size distribution, (**c**) FT-IR spectra, (**d**) XPS wide sweep and high resolution, and (**e**) C1, (**f**) N1 and (**g**) O1 spectra of S-PCDs.

S-PCDs were synthesized according to Cui et al. (2021) [16] and the synthesis process is shown in Figure 1a. Amounts of 2.5 g PEI and 2.5 g spermidine were added to 250 mL deionized water, and then transferred into polytetrafluoroethylene-lined hydrothermal reaction autoclave and heated at 220 °C for 3 h to form S-PCDs.

### 2.3. Characterization of S-PCDs

According to the method of Pan et al. (2022) [10], the morphology, surface chemical elements and functional groups of S-PCDs were characterized by transmission electron microscopy (TEM), X-ray photoelectron spectra (XPS) and FT-IR.

The liquid samples of S-PCDs were dropped onto a copper mesh and dried in a vacuum drying oven under dust-free conditions, and then electron microscope operating (H-700, Hitachi, Tokyo, Japan) at 200 kV was used to obtain TEM images of S-PCDs. Nano measurer 1.2 software was used to analyze the particle size of approximately 120 monodispersed S-PCDs in TEM images.

S-PCD solution was dripped onto the cover glass and dried in a drying oven. After this was repeated several times, a K-Alpha XPS system (Thermo Fisher Scientific, USA) was used to collect the XPS data of S-PCDs.

Freeze-dried S-PCDs powders were placed on infrared crystals of a Nicolet iS10 FT-IR spectrometer (Thermo Fisher, USA) and then the chemical functional groups of S-PCDs were identified. FT-IR spectra were recorded at 4 cm^−1^ resolution in a range of 4000–500 cm^−1^ against a background of ambient air.

### 2.4. Antibacterial Activity of S-PCDs

#### 2.4.1. MIC of S-PCDs against *S. aureus*

The 96-well microplate was used to determine the MIC [17]. S-PCDs diluted solution (256 μg/mL) was prepared in sterile broth. An amount of 200 μL S-PCDs was added to 1# well and LB medium (100 μL) was added to wells 2–10. Solution (100 μL) was removed from well 1 to well 2. After mixing, moved 100 μL solution from well 2 to well 3 and repeated the process to well 10. Then, 100 μL solution in well 10 was removed and discarded; the final solution was 100 μL per well. And then, each well was supplemented with 10^6^ CFU/mL of bacterial suspension. The final S-PCDs concentrations in wells 1–10 were 128, 64, 32, 16, 8, 4, 2, 1, 0.5 and 0.25 μg/mL. Sterile LB broth (200 μL) was added to wells 11–12 as control group.

#### 2.4.2. Bacteriostatic Circle and Bacteriostatic Rate

The antibacterial activity of S-PCDs against *S. aureus* was determined using Oxford cup drilling method. The bacterial suspension was mixed well with 25 mL of LB solid medium and poured into petri dish with an Oxford cup at the bottom. S-PCDs (180 μL) with 0, 1/4, 1/2, 1 and 2 MIC were added to the well formed by removing the Oxford cup. The diameters of bacteriostatic circles of 4 μg/mL PEI, spermidine and kanamycin against *S. aureus* were determined and the bacteriostasis rate analyzed as follows [18]:

Bacteriostasis rate (%) = (*D _sample_* − *D _control_*)/*D _sample_* × 100%

where *D _sample_* and *D _control_* are the diameters of the bacteriostatic circles produced with S-PCDstreatment and in the control group.

#### 2.4.3. Spread Plate Method to Determine Antibacterial Activity

Briefly, bacterial suspension was co-cultured with 0, 1/4, 1/2, 1 and 2 MIC S-PCDs at 37 °C for 24 h. Subsequently, 100 μL of diluted co-cultures were evenly spread on the LB nutrient agar and incubated at 37 °C for 24 h. The antibacterial activity of S-PCDs and 4 μg/mL PEI, spermidine and kanamycin was calculated by counting colony-forming units (CFUs) on plates.

#### 2.4.4. Bacteriostatic and Bactericidal Curves of *S. aureus*

The bacteriostatic and bactericidal curves of S-PCDs were determined [19]. For growth curve, the final concentration of *S. aureus* was diluted to 10^6^ CFU/mL. Then, the suspension of bacteria in each test tube was treated with S-PCDs of 0, 1/16, 1/8, 1/4, 1/2 and 1 MIC. The 180 μL of co-cultures was removed to a 96-well microtitration plate every 2 h and then the OD_600_ nm was measured using a microplate reader (Bio-Rad, Hercules, CA, USA) to monitor the growth of *S. aureus.*

As for time–kill kinetics analysis, the 10^6^ CFU/mL suspension of *S. aureus* was treated with S-PCDs of 1, 2, 4 and 8 MIC. After being further cultured at 37 °C for 24 h, the co-cultures (1 mL) were taken every 2 h. Then, dilution (100 µL) was evenly applied to LB agar plates and incubated; *S. aureus* suspension without S-PCD treatment was set as control.

### 2.5. Antibacterial Mechanism of S-PCDs

#### 2.5.1. The Damage of Membrane Permeability

Propyl iodide (PI) uptake test was determined [20]. The suspension of *S. aureus* was centrifugated at 6000× *g* for 15 min to obtain the bacterial cells, and they were re-suspended in 10 mL PBS. The 10^6^ CFU/mL of bacterial cells was treated with 0 and 1 MIC of S-PCDs for 24 h at 37 °C. The intensity of the co-culture was determined using a fluorescence spectrophotometer (Hitachi, Tokyo, Japan) after the reaction of PI in the dark at 37 °C for 10 min. The emission wavelength was set at 500–700 nm and the excitation wavelength was set at 495 nm.

#### 2.5.2. Determination of Extracellular Nucleic Acid and Protein Content

Nucleic acid assay was performed with reference to Wang et al. (2021) [21]. The *S. aureus* suspension was treated with 1 MIC of S-PCDs and cultured at 37 °C for 12 h. The co-culture was removed every 2 h and centrifuged for 15 min at 1000× *g*. The absorbance at 260 nm of supernatant was determined by a T9s UV–vis spectrophotometer (General Instrument Co. Ltd., Beijing, China).

The effects of S-PCDs on soluble proteins of bacteria were determined using a BCA kit. The *S. aureus* suspension was treated with S-PCD of 1 MIC and cultured for 12 h. The co-culture was removed and centrifuged at 1000× *g* for 15 min every 2 h. The BCA kit was used to measure the protein content.

#### 2.5.3. Microscope Observation of Morphology of *S. aureus*

The co-cultures of *S. aureus* suspension and 1 MIC S-PCDs were centrifuged at 500× *g* for 15 min to obtain the bacterial cells. After overnight fixation in glutaraldehyde (2.5%), the cells of *S. aureus* were washed again with PBS 3 times and dehydrated with ethanol solutions (30, 50, 70, 90 and 100%) for 15 min each. The morphology and surface characteristics of the strain were observed using SEM (Hitachi, Tokyo, Japan) after it was sputter-coated with gold.

The methods of bacterial culture, cleaning and fixation are the same as above. The bacterial suspension treated with SP-CDs was diluted to an appropriate concentration and adsorbed using a special copper mesh for TEM. The structure of fully dried *S. aureus* was observed using an H-700 TEM (Hitachi, Tokyo, Japan).

#### 2.5.4. Confocal Laser Scanning Microscopy Detected Bacterial Viability

*S. aureus* was stained with a L7012 Live/Dead bacterial staining kit [10]. The co-cultures of *S. aureus* suspension and S-PCDs were centrifuged at 10,000× *g* for 10 min to obtain the bacterial cells, then washed with 0.85% NaCl 3 times and re-suspended in 2 mL 0.85% NaCl. The bacterial cells (1 mL) were treated with 3 µL SYTO 9 and PI (SYTO 9:PI = 1:1) in a dark environment for 15 min. Fluorescent images were obtained using a CLSM (Leica TCS SP5, Heidelberg, Germany).

#### 2.5.5. Intracellular Reactive Oxygen Species (ROS)

S-PCDs of 1, 2 and 4 MIC were added into the 10^6^ CFU/mL of *S. aureus* suspension and co-incubated at 37 °C for 24 h. After centrifugation at 6000× *g* for 5 min, the bacterial cells were re-suspended in 10 mL PBS. The suspension was supplemented with 2′, 7′-dichlorodihydrofluorescein diacetate (10 μL, DCFH−DA, 10 μmol/L) and incubated for 30 min, and then the fluorescence intensity was detected. Bacteria without any treatment were used as a control group. The excitation wavelength was 484 nm and the emission wavelength was 525 nm. The ROS generation capacity was expressed by the relative ROS level [22]: *ROS _sample_*/*ROS _control_.*

#### 2.5.6. Gene Expression

The relative gene expression analysis was performed according to Zheng et al. (2021) [23]. Quantitative real-time polymerase chain reaction (qRT-PCR) was determined using the gene 9600 Quantitative PCR Instrument (Bori, Hangzhou, China). The expression of 3 indicator genes that can maintain intracellular redox balance, namely, 3 oxidation regulatory genes *dmpI*, *narJ* and *narK*, was detected after S-PCD treatment, and gyrB was used as the internal reference gene. Primers are shown in Table 1.

### 2.6. Antibiofilm Activity of S-PCDs

Firstly, the antibiofilm activity of S-PCDs was determined using crystal violet staining [24]. The final concentrations of S-PCDs were 0, 1/4, 1/2 and 1 MIC in the suspensions containing 200 μL *S. aureus*, and they were cultured for 24 h, the supernatant removed, and then they were rinsed with PBS 3–5 times and stained with 0.4% crystal violet for 5 min. After rinsing with sterile water and drying for 10 min, each well was supplemented with 200 μL 95% ethanol and OD_570_ was measured using an enzyme-labeled meter. Each sample was repeated three times.

Secondly, the spread plate method was used to measure the number of bacteria in the biofilm [25]. Amounts of 2 mL LB broth and 20 μL *S. aureus* suspension were added to 24-well plates with an aseptic coverslip at the bottom, and treated with 0, 1/4 MIC, 1/2 MIC and 1 MIC of S -PCDs at 37 °C for 24 h. Then, the coverslip was cleaned with PBS 3 times and then placed in LB broth for 2 min vortex to completely release the bacterial cells. The 10-fold dilutions of bacterial suspension were inoculated in LB agar to count the total number of bacterial colonies at 37 °C for 24 h.

The biofilm formation was observed using CLSM [26]. According to above method, bacteria were allowed to grow on the coverslip at 37 °C for 48 h to form biofilm. The biofilm formation was then detected by staining in the dark for 15 min with the L7012 LIVE/DEAD bacterial staining kit. A CLSM (Leica TCS SP5, Heidelberg, Germany) was used to record the bacterial fluorescent images.

### 2.7. Safety Evaluation of S-PCDs

CCK-8 method was used to evaluate the effects of S-PCDs on L929 cell viability and proliferation [5]. L929 cells were passaged in L929 medium at 37 °C in 5% carbon dioxide and 95% air. S-PCDs with different gradient concentrations (0, 2, 4, 8, 16, 32 and 64 μg/mL) were prepared using sterile L929 medium. After the cells were cultured on a 96-well plate until wall attachment, the medium was discarded and different concentrations of S-PCD solutions were added. After incubating again for 24 h, the cell cultures were incubated with 10% (*v*/*v*) CCK-8 solution for 3 h and the absorbance at 450 nm wavelength was measured to calculate the cell viability.

In addition, the cells treated with S-PCDs were stained with calcitanin AM and PI according to Dead/Live kit instructions. And a fluorescence microscope (Nikon A1 MP, Tokyo, Japan) was taken to obtain the corresponding fluorescence images.

### 2.8. Effects of S-PCDs on the Growth of S. aureus in Pasteurized Milk

The *S. aureus* cells were diluted to approximately 1 × 10^6^ CFU/mL with 20 mL of fresh pasteurized milk and incubated at 25 °C and 4 °C, after adding 1 MIC of S-PCDs. The control group did not contain S-PCDs. Microbial concentrations were determined using colony counting method after 1, 12, 24, 48 and 72 h of incubation. In addition, the pasteurized milk after 72 h of treatment was poured into a flat dish and left to stand for 30 min to observe the precipitation formed in the pasteurized milk [27].

### 2.9. Statistical Analysis

All experiments and their associated statistical analyses were conducted in triplicate and the results were expressed as mean ± standard deviation (SD). A significance level of 0.05 was used. The statistical analyses were performed using SPSS 26.0 (IBM SPSS Statistics, Chicago, IL, USA) software and the drawings were performed using Origin 2022b (Origin Lab Corporation, Northampton, MA, USA).

## 3. Results and Discussion

### 3.1. Performance Characterization of S-PCDs

S-PCDs can show a bright fluorescence emission phenomenon under a 365 nm violet lamp (Figure 1a), which indicated the successful synthesis of CDs by hydrothermal reaction. The S-PCDs were spherical particles with good dispersion without obvious aggregation (Figure 1b). The particle size of most S-PCDs ranged from 3.92 nm to 11.4 nm and the mean diameter of the S-PCDs was 6.87 nm. The diameter of carbon dots is usually less than 10 nm [28] and the diameter of the S-PCDs conforms to the typical particle size distribution range of carbon dots.

The absorption peak near 3277 cm^−1^ is related to the amino/amide N-H bond stretching vibration (Figure 1c). The characteristic absorption peaks of C−H appeared in the vicinity of 2944 and 2839 cm^−1^, and the peak at 1567 cm^−1^ was attributed to N−H deformation vibration. There were obvious C−H characteristic absorption peaks near 1466 cm^−1^, and the infrared absorption peaks at 1310 and 1145 cm^−1^ were related to the stretching vibration of C−O.

The surface chemical composition of S-PCDs was determined using XPS. The results of an XPS element sweep showed that the signal peaks at 284.1, 398.5 and 530.1eV of XPS binding energy were C1s, N1s and O1s respectively (Figure 1d). As the main elements of S-PCDs, the C, N and O relative contents were 76.79%, 11.31% and 11.9%, respectively. Because it is easy for the N-rich precursor to participate in the synthesis reaction, the content of the N element in the S-PCDs was high. Figure 1e shows the XPS high resolution spectrum of C1s. It can be seen that the main forms of the C element in S-PCDs were C−C/C=C (284.3–284.6 eV), C−N (285.4 eV) and C=O/C=N (287.3 eV) [29]. C−C/C=C is the main component of S-PCDs’ skeleton, which is related to intramolecular dehydration. In addition, according to the C1 peak splitting results, the peak splitting area of XPS related to the C=N/C=O bond was small, indicating that the S-PCDs contained only a small amount of carbonyl groups on the surface, while the peak splitting area of XPS related to the C−N bond was large, indicating that spermidine can effectively participate in the formation of CD carbon nuclei. The N1 spectrum in Figure 1f shows the XPS signal peaks of 398.4 eV and 399.0 eV. There is no peak above 401.0 eV, indicating that the sample is without the N−N bonding structure [29]. The main peak of 398.4 eV usually belongs to the sp^2^ N atom, while the signal peak at 399.0 eV was assigned to bridging N atoms in N−(C)_3_ or N bonded with H atoms [30,31]. From the O1 spectrum (Figure 1g), it can be seen that the main forms of the O element in S-PCDs were C=O (530.8 eV), C−OH/C−O−C (531.2 eV) and C−O (534.6 eV) [31].

### 3.2. Antibacterial Activity of S-PCDs

**Figure 2:**
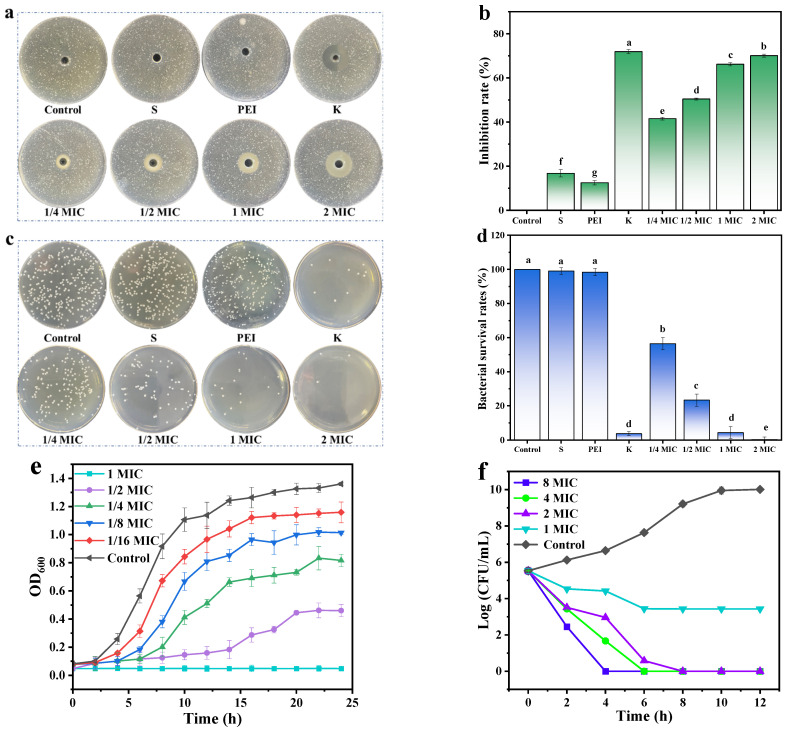
Antibacterial activity of S-PCDs. (**a**) Inhibition zone diameter, (**b**) inhibition rate, (**c**) antibacterial activity, and (**d**) bacterial survival rates of S-PCDs on *S. aureus*. *S. aureus* was treated with different concentrations of S-PCDs. The same concentrations of S and PEI as S-PCDs were used as negative control, and kanamycin was used as positive control. (**e**) *Bacteriostatic curve* and (**f**) bactericidal curve of different concentrations of S-PCDs against *S. aureus*. The average values of different letters in (**b**,**d**) were significantly different when *p* < 0.05.

The MIC of S-PCDs against *S. aureus* was 16 μg/mL. S-PCDs and kanamycin showed clear inhibition circles with diameters of 22.2 and 27.3 mm, respectively, while spermidine and polyethyleneimine showed almost no visible inhibition circles (Figure 2a). Furthermore, the diameter of the inhibition circles and the inhibition rate increased significantly (*p* < 0.05) with increasing the S-PCD concentration. When treated with 2 MIC S-PCDs, the inhibition circle diameter reached 25.8 mm and the inhibition rate of 70.11% was not significantly different from that of kanamycin (71.97%) (Figure 2b).

The number of colonies of *S. aureus* did not change after spermidine and polyethyleneimine treatment (Figure 2c). The bacterial viability tended to zero when S-PCDs were treated at 1 MIC and higher concentrations, and there was a significant decrease in the total number of colonies and a significant increase in bacterial viability as the concentration of S-PCDs increased (Figure 2d).

The time-dependent bacteriostatic curve was determined by measuring the OD_600_ of the co-cultures of 1/16−1 MIC S-PCDs and *S. aureus* suspension (Figure 2e). The OD_600_ of *S. aureus* increased between 4 and 10 h, and the OD_600_ decreased significantly after treatment with S-PCDs in different concentrations. As the concentration of S-PCDs gradually increased from 1/16 MIC to 1 MIC, the inhibition curve gradually decreased with time. The concentration-dependent inhibition of S-PCDs was consistent with the results of the inhibition circle assay.

The bactericidal curve of S-PCDs on *S. aureus* showed that *S. aureus* untreated with S-PCDs grew rapidly at 4–8 h of incubation (Figure 2f). When *S. aureus* was treated with MIC and higher S-PCD concentrations for 12 h, the total number of colonies showed a continuous decreasing trend with time. In addition, as the concentration of S-PCDs increased, the total number of colonies decreased in the same treatment time. Two MIC S-PCDs could almost kill *S. aureus* within 8 h and 4 MIC S-PCDs could almost kill *S. aureus* within 6 h. The results demonstrated that S-PCDs had good bacteriostatic activity against *S. aureus* in a concentration-dependent manner.

### 3.3. Cell Membrane Damage of S. aureus

**Figure 3:**
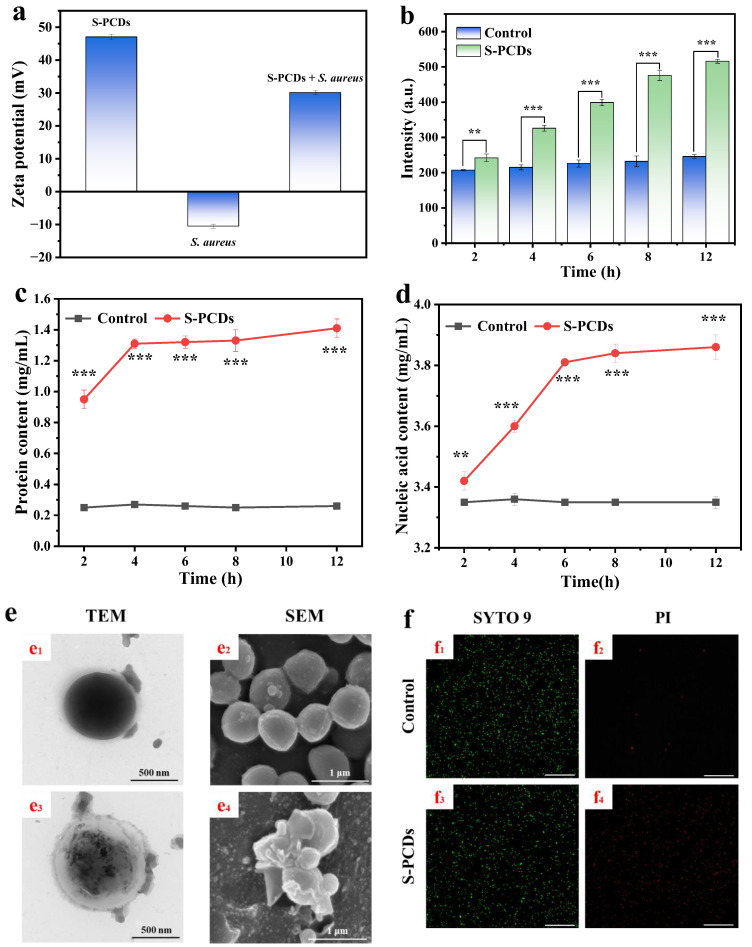
Bacterial cell membrane damage. (**a**) Zeta-potential of S-PCDs, *S. aureus* (with or without S-PCD treatment). (**b**) Uptake of PI in *S. aureus*. Effect of S-PCDs on (**c**) the extracellular protein content and (**d**) the extracellular nucleic acid content of *S. aureus*. (**e**) TEM and SEM images of *S. aureus*. *S. aureus* without treatment (e_1_, e_2_, f_1_ and f_2_) or with treatment of S-PCDs (e_3_, e_4_, f_3_ and f_4_). (**f**) CLSM images of *S. aureus* were stained by SYTO 9/PI dual fluorescence staining. All bacteria alive and dead were stained by SYTO 9 showing green fluorescent intensity. Only dead bacteria can be stained by PI emitting red fluorescent intensity. For SYTO 9: λex = 488 nm; for PI: λex = 552 nm. The scale bar is 75 μm. ** *p* < 0.01, *** *p* < 0.001.

The zeta potentials of S-PCDs and *S. aureus* were +47.06 and −10.5 mV (Figure 3a). The zeta potential of *S. aureus* increased to +30.14 mV after treatment with S-PCDs. The results suggested that the high positive charge of the S-PCDs positively shifts the negative charge of *S. aureus* and electrostatic interaction may occur between the two. This electrostatic interaction may cause CDs to further destroy the surface charge and physiological metabolic activities of *S. aureus*, eventually leading to bacterial death [17].

PI can only enter the interior of the cells to bind nucleic acids to produce a fluorescence signal after the bacterial membrane is broken. The PI uptake of the S-PCD treatment group increased significantly with incubated time, and the fluorescence intensity reached 2.1 times that of the control group after culturing for 12 h (Figure 3b). The fluorescence intensity of the control group remained unchanged. The results suggested that S-PCDs can alter the permeability of cell membranes and cause bacterial lysis, allowing PI to enter the cell interior and bind to DNA.

The extracellular protein content of *S. aureus* after S-PCD treatment was significantly increased (*p* < 0.001) (Figure 3c). OD_260_ nm increased rapidly after culturing for 2–6 h and gradually stabilized after culturing for 8 h (Figure 3d). The extracellular protein and nucleic acid content were 1.41 and 3.86 mg/mL, respectively, which were 5.4 and 1.2 times those of the control group when cultured for 12 h. The results indicated than S-PCDs could disrupt the integrity of the membrane and lead to a significant leakage of cytoplasmic contents from bacterial cytoplasm. Leakage of cytoplasmic contents such as DNA, RNA and proteins is an indicator of irreversible damage to bacterial membranes [21].

The damage of S-PCDs to *S. aureus* was further evaluated using TEM and SEM. TEM images showed that the untreated *S. aureus* grew well, and its cell membrane had a complete, smooth and clear morphological structure (Figure 3e_1_). After S-PCD treatment, the cell wall and membrane of *S. aureus* became irregular, and the cells became transparent, accompanied by intracellular leakage (Figure 3e_2_). In addition, untreated cells showed complete spherical structures with clear edges and smooth cell walls (Figure 3e_3_). However, after 24 h treatment with S-PCDs, many membrane defects and leakage of cell contents were found on the bacterial cell surface (Figure 3e_4_), indicating that the bacteria were damaged. The results of SEM and TEM demonstrated that the adsorption of S-PCDs on the bacterial wall or outer membrane caused physical or mechanical damage to it, resulting in the overall physiological dysfunction of the bacteria and even the leakage of cytoplasmic components, and finally led to the death of bacteria.

The DNA of all live and dead bacteria can be stained by SYTO 9, while PI can only stain bacteria with damaged cell membranes [32]. Both the S-PCD treated group and the control group stained by SYTO 9 showed a large amount of green fluorescence. The red fluorescence of the S-PCD treatment group was significantly enhanced compared with the control group, indicating that a large number of dead bacteria were induced to emit red fluorescence after S-PCD treatment, which further confirmed the effective bactericidal ability of S-PCDs.

### 3.4. ROS Generation of S. aureus

**Figure 4:**
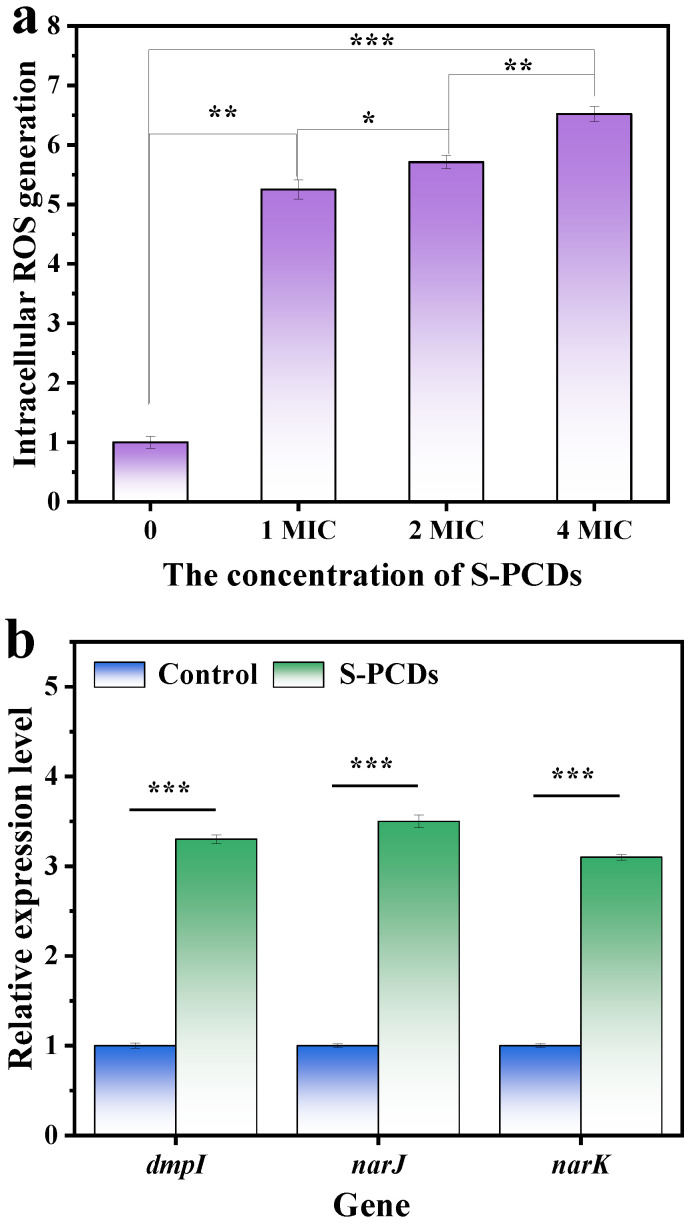
ROS generation in *S. aureus*. (**a**) The generation of ROS in *S. aureus* treated with S-PCDs by using the dye DCFH-DA. (**b**) Comparison on relative expression level of *dmpI*, *narJ* and *nark* in *S. aureus* after treatment with S-PCDs. * *p* < 0.05, ** *p* < 0.01. *** *p* < 0.001.

The results of ROS production are shown in Figure 4a. The fluorescence intensity of *S. aureus* was significantly stronger than that of the control group after treatment with 1–4 MIC S-PCDs, indicating that S-PCDs stimulated ROS production. After 1 MIC concentration of S-PCDs treatment, the ROS production of *S. aureus* was 5.25 times that of the control group. And the fluorescence intensity of *S. aureus* treated with S-PCDs was significantly increased with increasing concentration of S-PCDs (*p* < 0.05). When treated with 4 MIC of S-PCDs, the ROS production reached 6.52 times that of the control group. Many nanoparticles exhibit high antibacterial activity against a variety of pathogens, including drug-resistant bacteria, and the antibacterial mechanism is through increasing physical damage to cell membranes. In addition, they can exert antibacterial activity through photoinduction or thermal induction of free radicals and ROS [33]. This study showed that, after the interaction between CDs and bacteria, ROS production can be induced through light-independent reactions, so that ROS can induce oxidative stress and destroy bacterial cell membranes to produce antibacterial effects.

The nanomaterials can contribute to the disruption of the metabolic balance of intracellular oxidation reactions by upregulating genes encoding oxidation-stimulating enzymes (such as *dmpI*, *narJ* and *nark*) to produce large amounts of ROS [22]. The expression of three indicator genes, namely the three oxidative regulatory genes *dmpI*, *narJ* and *narK*, which can maintain intracellular redox balance, was detected after S-PCD treatment. The *dmpI* gene encodes 4-oxalocrotonate tautomerase, which participates in the oxidative catabolism of toluene, o-xylene, 3-ethyltoluene and 1,2, 4-tritoluene into corresponding intermediates in the citric acid cycle, and produces ROS as a by-product. The *narJ* and *narK* genes are important participants in respiratory nitrate reductases, which transfer electrons from NADH or NADPH to nitrate, assisting in nitrate transport and nitrate reduction [34]. The results showed that treatment with S-PCDs could induce the expression of three oxidation genes to be significantly upregulated to 3.3, 3.5 and 3.1 times (*p* < 0.001), respectively (Figure 4b), which was also consistent with the data generated by ROS. Since ROS is a by-product of intracellular redox metabolism, the difference in ROS production in the S-PCD treatment group may be due to the different redox reactions of bacteria to them [23]. Oxidative stress-induced lipid peroxidation leads to cell membrane rupture, which causes DNA degradation induced by oxidative stress after CDs enters bacterial cells, resulting in bacterial death [35].

### 3.5. Antibiofilm Activity of S-PCDs

**Figure 5:**
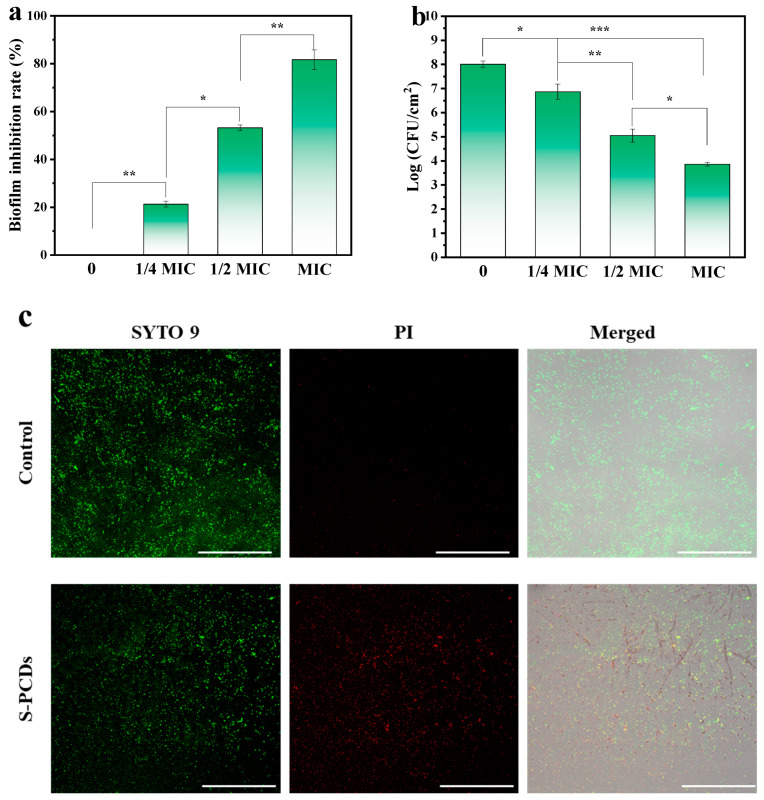
Antibiofilm activity of the S-PCDs. (**a**) Effects of S-PCDs on biofilm formation of *S. aureus*. (**b**) The number of viable bacteria from biofilms on coverslips surfaces. (**c**) CLSM images of *S. aureus* were stained by SYTO 9/PI dual fluorescence staining. All bacteria alive and dead were stained by SYTO 9 showing green fluorescent intensity. Only dead bacteria can be stained by PI emitting red fluorescent intensity. For SYTO 9: λex = 488 nm; for PI: λex = 552 nm. The scale bar is 250 μm. * *p* < 0.05, ** *p* < 0.01, *** *p* < 0.001.

The inhibition rates of S-PCDs with 1/4, 1/2 and 1 MIC were 21.34%, 53.42% and 81.34%, respectively. The compact structure of the biofilm protects the bacteria from antibacterial substances. Therefore, the viable bacteria number in the biofilm formed after S-PCD treatment was measured. S-PCDs showed effective inhibition in a dose-dependent manner. The number of living bacteria was reduced to 3.89 log CFU/cm^2^ when treated with 1 MIC S-PCDs (Figure 5b). CLSM explored the effect of S-PCDs on *S. aureus* biofilm, and the results are shown in Figure 5c. The green fluorescence intensity decreased and the red fluorescence intensity increased significantly after 1/2 MIC S-PCD treatment compared with the control group. S-PCDs inhibited the biofilm of *S. aureus* by reducing the cell viability and the number of viable bacteria.

### 3.6. Safety Evaluation of S-PCDs

**Figure 6:**
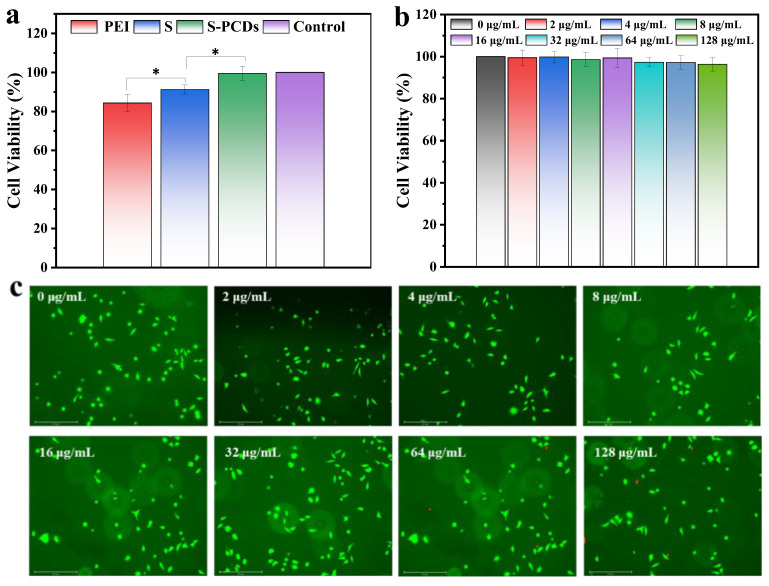
Safety evaluation of S-PCDs. (**a**) Cell viability of L929 fibroblasts treated with PEI, S and S-PCDs (2 μg/mL) for 24 h. (**b**) Cell viability of L929 fibroblasts treated with different concentrations of S-PCDs (0, 2, 4, 8, 16, 32, 64 and 128 μg/mL) for 24 h. (**c**) Live/Dead fluorescence staining images after 24 h incubation. * *p* < 0.05.

The cytotoxicity of S-PCDs on L929 cells was determined using a CCK-8 kit. As shown in Figure 6a, when 2 μg/mL of spermidine, polyethyleneimine and S-PCDs was added to the medium and cultured for 48 h, cell viability was >80% in all groups. However, the cell viability of S-PCDs was significantly enhanced compared to that of spermidine and polyethyleneimine, thus further improving safety, which is consistent with the reduced toxicity of synthetic carbon dots reported by most investigators. The cell viability of S-PCDs at 0–128 μg/mL was >95% after 24 h of incubation (Figure 6b). In addition, L929 cells treated with 2–128 μg/mL S-PCDs proliferated normally and showed similar cell morphology to the control group, with almost no dead cells detected (Figure 6c). The results indicated that S-PCDs have excellent biocompatibility and a better safety profile.

### 3.7. Effects of S-PCDs on Growth of S. aureus in Pasteurized Milk

**Figure 7:**
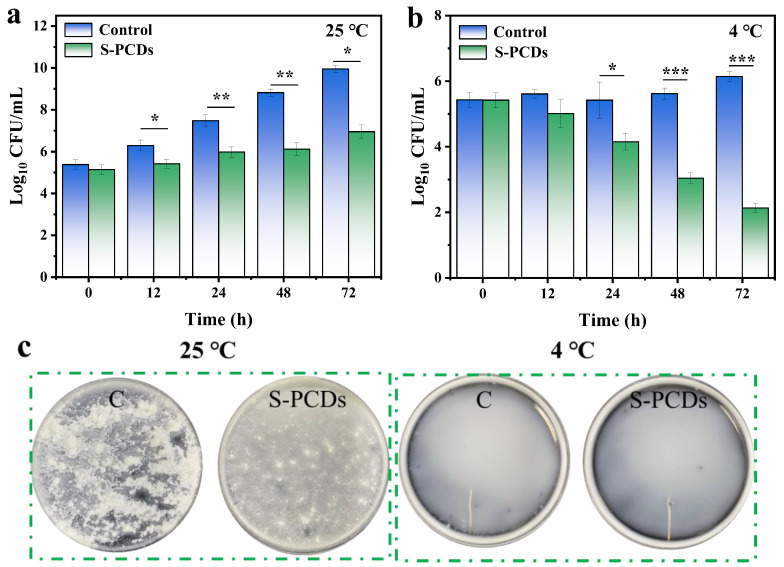
S-PCDs on the growth of *S. aureus* in pasteurized milk. The mode of *S. aureus* infected with pasteurized milk when treated with nisin and CAR used alone or in combination at (**a**) 25 °C and (**b**) 4 °C, and (**c**) the formation of precipitation in treated pasteurized milk. * *p* < 0.05, ** *p* < 0.01, *** *p* < 0.001.

The results of pasteurized milk treated with S-PCDs after *S. aureus* infection are shown in Figure 7. When the pasteurized milk was stored at 25 °C for different times, S-PCDs inhibited the growth of *S. aureus* (Figure 7a). After 24 h and 72 h treatment with S-PCDs, the concentration of *S. aureus* decreased from 7.48 to 5.98 log_10_ CFU/mL and 9.95 to 6.95 log_10_ CFU/mL, respectively (*p* < 0.01). The results showed that S-PCDs had a significant inhibitory effect on *S. aureus* in pasteurized milk. *S. aureus* in pasteurized milk survived and grew normally at 4 °C for 72 h (Figure 7b). The antibacterial activity of S-PCDs was significantly different after 24 h compared with the control group (*p* < 0.05). Compared with the control group, the number of microorganisms was significantly decreased after S-PCD treatment for 72 h (*p* < 0.001). This indicated that S-PCD treated milk can be stored for a long time. The morphology of the treated pasteurized milk sample was then observed by laying it on a flat plate. After growing *S. aureus* at 25 °C for 72 h, a large amount of precipitation was observed in all samples (Figure 7c), accompanied by a strong rancid smell. Since proteins and lipids in milk are agglomerated under the growth and metabolism of microorganisms, S-PCD treatment can effectively reduce the formation of precipitation, which means that its growth and metabolism are inhibited.

## 4. Conclusions

In summary, S-PCDs were synthesized and characterized by hydrothermal synthesis. The antibacterial activity, antibacterial mechanism and antibiofilm activity of S-PCDs were systematically studied using *Staphylococcus aureus* as a model. The results showed that the antibacterial activity of S-PCDs was stronger than that of the two raw materials. The high positive charge and small size of the surface of S-PCDs enable them to inhibit *S. aureus* by damaging the membrane and producing ROS. In addition, S-PCDs can effectively inhibit the formation of biofilms. Safety evaluation results showed that S-PCDs had low cytotoxicity. S-PCDs inhibited the number of microorganisms in the pasteurized milk infected with *S. aureus* and maintained the quality of the pasteurized milk at 4 °C and 25 °C. This study clearly elucidates the interaction between S-PCDs and *S. aureus*, provides new insights into the role of carbon points in food, and further supports the in-depth application of nanomaterials.

## Figures and Tables

**Table 1:** RT-PCR primers.

Gene	Forward Primer 5′ to 3′	Reverse Primer 3′ to 5′
*dmpI*	TGATGCCAATCGTCAATG	CCCGTTGTTTTTTCTACG
*narJ*	GAACGTGGGCAAATGTTAG	TTGAAGCATCAACGGTAG
*narK*	TATTCCCGATATTTTTCTTAAGCC	CTGATGTAACACCAACAGAG
*gyrB*	TCAATACAGGTTTTAGAGGGGTTA	AACCATTCAATACTTCATCGACG

## Data Availability

Data is contained within the article.
